# The TRPA1 Channel Mediates Mechanical Allodynia and Thermal Hyperalgesia in a Rat Bone Cancer Pain Model

**DOI:** 10.3389/fpain.2021.638620

**Published:** 2021-03-22

**Authors:** Qiangwei Liu, Long Feng, Xiujing Han, Weidong Zhang, Hong Zhang, Longhe Xu

**Affiliations:** ^1^Department of Anesthesiology and Operation, The First Medical Center of Chinese PLA General Hospital, Beijing, China; ^2^Department of Anesthesiology, Hainan Hospital of Chinese PLA General Hospital, Sanya, China; ^3^Clinical Laboratory, The First Affiliated Hospital of Guangzhou Medical University, Guangzhou, China; ^4^Department of Anesthesiology, The Fifth Medical Center of Chinese PLA General Hospital, Beijing, China; ^5^Department of Anesthesiology, The Third Medical Center of Chinese PLA General Hospital, Beijing, China

**Keywords:** transient receptor potential ankyrin 1, TRPA1 antisense oligodeoxynucleotide, bone cancer pain, transient receptor potential, paw mechanical withdraw threshold, paw thermal withdraw latency, A967079, bone cancer pain model

## Abstract

**Background:** Bone cancer pain (BCP) significantly affects patient quality of life, results in great bodily and emotional pain, and creates difficulties in follow-up treatment and normal life. Transient receptor potential ankyrin 1 (TRPA1) is an essential transduction ion channel related to neuropathic and inflammatory pain. However, the role of TRPA1 in BCP remains poorly understood. This study aimed to explore the relationship between TRPA1 and BCP.

**Methods:** A BCP model was induced by Walker256 cells to the left tibia. The sham group was induced by normal saline to the left tibia. Thereafter, pain behaviors and TRPA1 expression between the BCP group and the sham group were observed on the 14th day of modeling. The TRPA1 antagonist A967079 (10 mg/kg) was injected via tail vein. TRPA1 antisense oligodeoxynucleotide (AS-ODN, 5 nmol/10 μl) and missense oligodeoxynucleotide (MS-ODN, 5 nmol/10 μl) were intrathecally delivered via a mini-osmotic pump for 5 consecutive days to assess the effect of TRPA1 on BCP. Behavioral tests were assessed preoperatively and postoperatively. Real-time quantitative PCR and western blot analyses were used to measure TRPA1 levels among the different groups.

**Results:** The BCP model was successfully established via X-ray and pathological sections at 14 days. Compared to the sham group, the BCP group was more sensitive to mechanical stimuli, cool stimuli and hot stimuli. Intravenously injected A967079 can relieve paw mechanical withdrawal threshold and paw withdrawal thermal latency in rats with BCP. Moreover, AS-ODN can relieve paw mechanical withdrawal threshold and paw withdrawal thermal latency in rats with BCP. Additionally, relative mRNA and protein expression of TRPA1 in the BCP group were much higher than those in the sham group (14.55 ± 1.97 vs. 1 ± 0.04, *P* < 0.01). Compared to the BCP group, the relative mRNA and protein expression of TRPA1 in the BCP+AS-ODN group was reduced (14.55 ± 1.97 vs. 2.59 ± 0.34, *P* < 0.01).

**Conclusions:** The TRPA1 channel mediates mechanical allodynia and thermal hyperalgesia in a rat BCP model.

## Introduction

Since numerous advanced medicines and technologies have been explored and used for cancer treatment, patients with tumors exhibit longer survival times [1, 2]. Unfortunately, ~75–90% of patients continue to experience bone cancer pain (BCP), which significantly lowers their quality of life [1–5]. Some patients even suffer pain resulting from chemotherapeutic treatments [6]. Despite advances in medical technology, the mechanism of BCP remains unclear. BCP is a complex syndrome involving the complex interplay of mechanisms in the peripheral and central nervous systems [7–9]. At a minimum, the biology of BCP involves tumor cells, peripheral nerves and bone cells [10, 11]. A variety of noxious secretions and neurotransmitters change the micro-environment around tumors and bones, while specialized sensory neurons detect environmental stimuli and convert them to the central nervous system, which causes the sensation of pain [12]. BCP is a progressive pain state. Notably, over 50% of cancer patients have inadequate and undermanaged pain control [13]. Current therapies are insufficient for antinociceptive tolerance and disease-related pain progression. Thus, there exists an urgent need to find a new target for BCP and improve quality of life for patients with advanced cancer [5]. Transient receptor potential ankyrin 1 (TRPA1) ion channel is expressed in a subset of the nociceptive sensory neuron. It belongs to the transient receptor potential superfamily (TRPs), which includes non-selective, calcium-permeable cation channels activated by chemical stimuli, mechanical stimuli, and thermal changes [14]. TRPA1 detects environmental and endogenous chemical irritants such as isothiocyanate and cinnamaldehyde and is specifically sensitive to cool stimulation (< 17°C) [15]. TRPA1 also serves important roles in inflammation, neuropathic pain, chemotherapeutic pain and other forms of pain [12, 16, 17]. However, few studies have investigated whether TRPA1 is involved in BCP.

In this experiment, we established a reliable BCP model on Sprague-Dawley (SD) rats to detect the level of TRPA1 between a sham group and BCP group. Thereafter, two interventions were applied to antagonize TRPA1 and prove the relationship between TRPA1 and BCP.

## Materials and Methods

### Animals

A total of 52 female SD rats (ordered from Beijing Huafukang Biotechnology Co., Ltd., China), weighing 180–200 g, were raised in the Experimental Animal Center of the Chinese People's Liberation Army (PLA) General Hospital. All procedures involving animals were performed in accordance with the guide for the Care and Use of Laboratory Animals. The environment was maintained at 22–24°C, with 55% humidity, a 12-h light/dark cycle and free access to food and water.

The experiment was divided into two parts. In the first part, 10 rats were used as bone cancer pain models and randomly divided into two groups: BCP + normal saline (BCP, *n* = 5) and BCP + A967079 (*n* = 5). All drugs were injected intravenously at 9 am on the 7th postoperative day only (A967079, TRPA1 antagonist, 10 mg/kg, 10 μl; normal saline 10 μl) [18, 19].


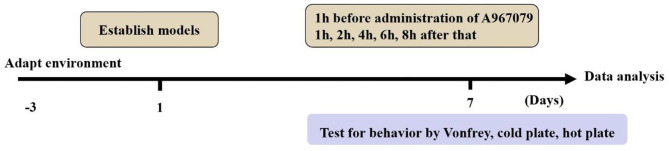


In the second part, 42 rats received intrathecal catheterisation. A total of six rats failed intrathecal catheterisation and were excluded. Thereafter, 36 rats with intrathecal catheterisation were randomly divided into four groups: sham + normal saline (sham+NS group, *n* = 9); BCP + normal saline (BCP+NS, *n* = 9); BCP + TRPA1 antisense oligonucleotide (BCP+AS-ODN, *n* = 9); BCP + missense oligonucleotide (BCP+MS-ODN, *n* = 9). AS-ODN or MS-ODN (5 nmol/10 μl) and NS (10 μl) were injected intrathecally via a mini-osmotic pump at a rate of 1 μl/h for 5 days [20]. The sample size of this study was mainly based on the pre-experiment results. The detection level was α = 0.05, β = 0.2 and the sample size was 7–10 in each group.


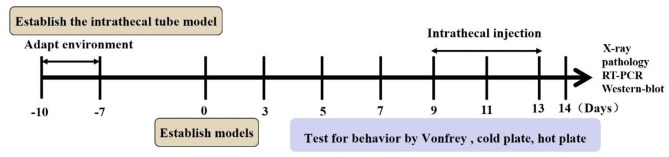


### Reagents and Apparatus

A967079 (Abcam, 144580, UK), AS-ODN (TRPA1 antisense oligodeoxynucleotide), TCTATGCGGTTATGTTGG (Invitrogen, Shanghai, China), MS-ODN (TRPA1 missense oligodeoxynucleotide), ACTACTACACTAGACTAC (Invitrogen, Shanghai, China), von Frey (North Coast Medical, Inc., Morgan Hill, CA, USA), Cool/Hot Plate Analgesiometer (Ugo Basile, Milan, Italy), PE-10 (Smiths Medical International Lt.Company,UK), TRPA1 antibody (Novus, NB110-40763SS, USA).

### Model Establishment

For the BCP model, suspensions of 1 × 10^4^/ml Walker256 tumor cells in PBS were prepared as previously described [12, 21]. The cells were placed on ice for preparation. After the rats were anesthetized with 1% sodium pentobarbital (IP 45 mg/kg), hair shaving and conventional disinfection were performed on the left hind limb and a 0.5 cm incision was then made on the upper tibia. Then, tissues and muscles were bluntly separated and part of the tibia surface was exposed. Prepared Walker256 tumor cells 10 μl were then slowly injected into the left tibia cavity of each rat using a 10 μl micro-injection syringe. The syringe was left in place for an additional 2 min to prevent the tumor cells from leaking out along the injection track. The injection site was closed by bone wax while the syringe was removed (to prevent the overflow of tumor cells) and then sutured layer by layer. The sham group rats were injected with PBS instead of Walker256 tumor cells. Rats were allowed to recover and were monitored daily.

For intrathecal drug administration, intrathecal catheterisation was implanted as previously described [22, 23]. Rats were anesthetized with 1% sodium pentobarbital (IP 45 mg/kg) and their back hair was shaved from approximately L1 to L6. Conventional disinfection was performed on the shaved area. Each rat was in the prone position with its abdomen padded up with an empty 60 ml syringe to arch the rat back. A 1-cm incision was made at the line of the vertical anterior superior iliac spine and the tissue and muscle were then bluntly separated. A sterilized PE-10 catheter filled with normal saline was inserted into the L5–L6 intervertebral space and the tip of the catheter was placed at the level of the lumbosacral enlargement. This was the correct placement due to rats' tail-flicking behavior. Then, the externalized portion through the skin was sutured at the neck and sealed effectively. The rats recovered for 7 days after intrathecal catheterisation. Rats were reserved if their lower limbs were paralyzed after being injected with 20 μl of 2% lidocaine through the catheter and recovered to normal ~20 min later. Conversely, rats were excluded if there was no reaction. Then, 80,000 u of penicillin was injected into the muscle after each surgery to prevent infection (**Figure 3**).

### Behavioral Tests

Paw mechanical withdrawal threshold (PMWT) was stimulated by von Frey hairs and calculated by the “up and down” method as previously described [22, 24, 25]. Before the test, rats were placed in individual plastic boxes with a wire mesh floor and habituated to the environment for 30 min. Monofilament was applied perpendicularly to the middle surface of the left plantar with a sufficient force to slightly bend the filaments for 6–8 s. The monofilament started from 2 to 60 g until the paw withdrew. A positive response was noted if the paw was sharply withdrawn or there was flinching upon removal of the hair. If no response was noted, a higher-force hair was tested. Each trial was repeated three times at ~5-min intervals.

Paw withdrawal thermal latency (PWTL) was assessed with a hot/cool plate analgesiometer as previously described [26]. The rats were allowed to habituate themselves to the testing environment for 30 min. For the cool plate test, the rats were kept individually in the center of a cool plate maintained at 0 ± 0.5°C for a maximum of 300 s. For the hot plate test, the rats were kept in a hot plate maintained at 52 ± 0.5°C for a maximum time of 30 s. If escape behaviors (e.g., lifting, flicking, or licking of hind paws) occurred before the maximum time, the rats were immediately removed from the apparatus. The average latency for cool or hot was calculated using three repeated trials at ~10-min intervals.

In the A967079-related groups, behavioral tests were performed before the rats were intravenously injected with A967079 or normal saline and at 2, 4, 6, and 8 h after injection. Behavioral tests for other groups were performed preoperatively 1 (T1) and at 3 (T3), 5 (T5), 7 (T7), 9 (T9), 11 (T11), 13 (T13), 14 (T14) days post-operatively.

### Assessment of the Bone Cancer Pain Model

To evaluate whether or not the BCP model was successfully established, X-ray imaging and pathological section were performed 14 days after tumor cells were implanted. X-ray examination (Faxitron RX-650) was used for small animal imaging to detect the loss of medullary bone and destruction of cortical bone in the tibia of the left hind limb. Animals with medullary bone loss and the erosion of cortical bone observed in the tibia were prepared for further experimentation. Pathological sections were performed to detect whether the bone cancer successfully developed. The dorsal root ganglion (DRG) of each rat was harvested, rinsed in PBS, fixed in 4% paraformaldehyde for 24 h, embedded in paraffin and cross-sectioned into 10-mm slices. Sections were then stained with hematoxylin and eosin (HE) for cell alignment.

### Quantitative Real-Time Reverse Transcriptase-Polymerase Chain Reaction

The left L4–6 rat DRG tissue was rapidly removed at 14 days post-operatively, immediately placed in liquid nitrogen and stored at −80°C until use. Trizol reagent (Thermo Fisher Scientific, Beijing, China) was used to extract total RNA. RNA concentration was then measured using a spectrophotometer. Thereafter, 500 ng of RNA was used to synthesize cDNA using a ReverTra Ace qPCR RT Master Mix with gDNA Remover (Toyobo, Beijing, China). Quantitative real-time PCR (qRT-PCR) was then performed using a PowerUp SYBR Green Master Mix Reaction Kit (Applied Biosystems, Beijing, China) according to the manufacturer's protocol and an ABI Prism 7900 sequence detection system (Applied Biosystems, Foster City, CA) was used. The primers for qRT-PCR reactions were as follows: TRPA1 primers (upstream primer: GCAGCATTTTCAGGTGCCAA, downstream primer: CGCTGTCCAGGCACATCTTA); GAPDH primers (upstream primer: GTTACCAGGGCTGCCTTCTC, downstream primer: GGGTTTCCCGTTGATGACC). The expression level of target mRNA was quantified relative to the level of GAPDH using the 2-ΔΔCT method.

### Western Blot Assay

Rats were anesthetized with 1% sodium pentobarbital (IP 45 mg/kg). The left L4–L6 DRG were rapidly harvested 14 days after surgery (*n* = 9 per group) and placed in liquid nitrogen. Western blot was used to analyse the protein expression levels of TRPA1 in DRG from different groups. The DRG tissues were mixed in RIPA lysis buffer and protease and phosphatase inhibitors, homogenized, then incubated on ice for 30 min. The homogenates were centrifuged at 12, 000 × g for 15 min at 4°C. The BCA method was used to measure the protein concentrations of the lysate. Before being transferred to a 0.2 mm polyvinylidene difluoride membrane, we loaded samples (10 ug of protein/lane) on a 10% SDS-polyacrylamide electrophoresis gel for 30 min at 80 V,then 60 min at 120 V and blocked for 1 h. The primary antibodies (rabbit anti-TRPA1, 1:500, Novus, NB110-40763SS; mouse anti-β-actin, 1:3,000, Protein Tech) were incubated with PVDF membranes overnight at 4°C. Goat anti-rabbit secondary antibody (IRDye680RD1; 5,000) and goat anti-mouse antibody (IRDye800CW1; 5,000) were incubated with the membranes at room temperature for 1 h. An enhanced chemiluminescence kit was used to visualize the protein bands. The densities of the detected proteins were analyzed by Image-Pro Plus Software and normalized to β-actin.

### Statistical Analysis

All data were analyzed by an observer who was blind to the experimental protocol. Statistical calculations were performed using the statistical analysis software GraphPad Prism, Version 6.0 (GraphPad, San Diego, CA, USA). Data were expressed as the mean ± standard deviation (SD). In the first part, behavioral experimental data were analyzed by *T*-test. In the second part, data were analyzed by Two-way ANOVA test, followed by the Tukey's multiple comparison test. When *P* < 0.05, the difference was considered statistically significant.

## Results

### Establishment of the BCP Model

**Figure 1:**
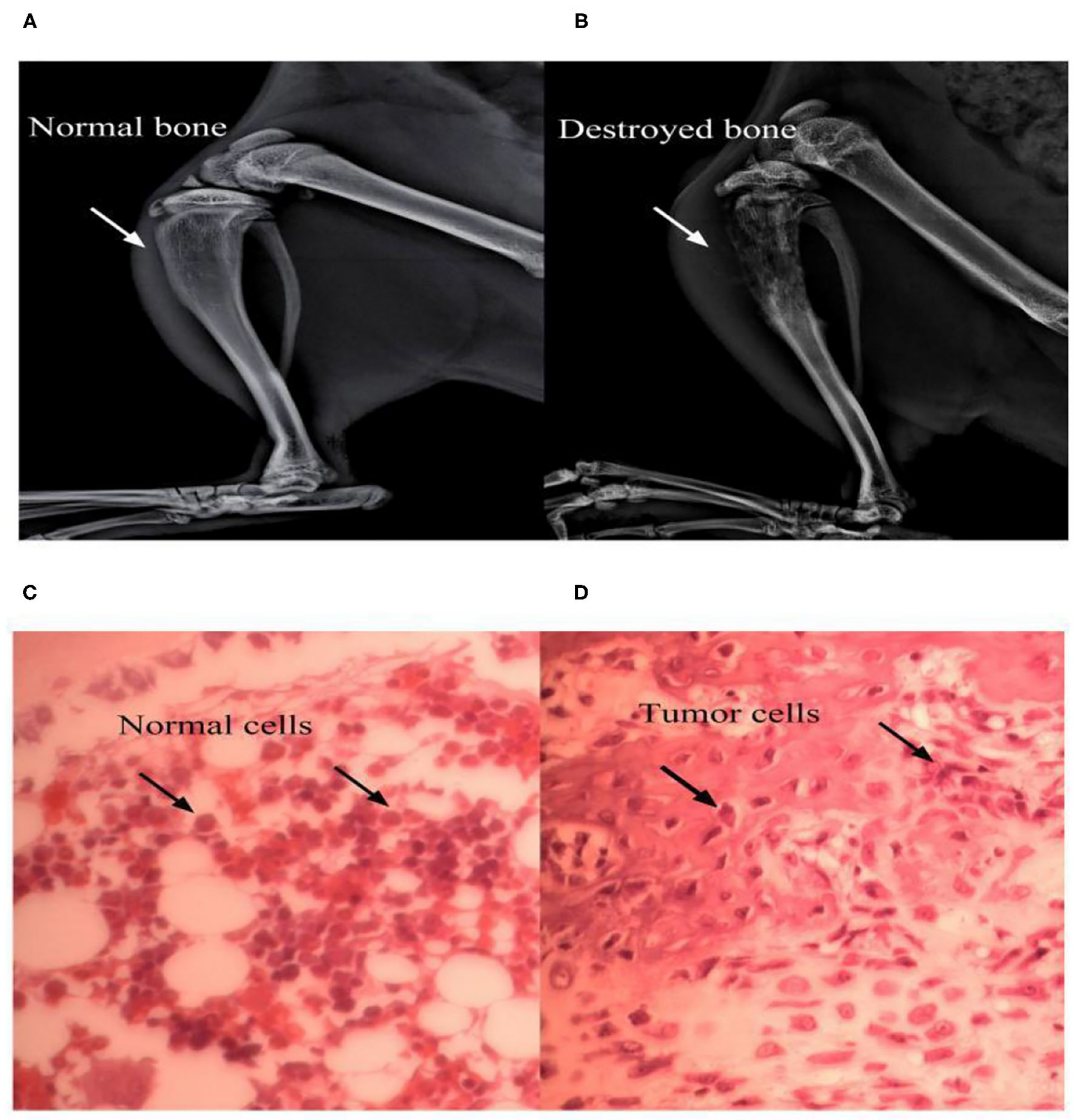
Bone destruction at 14 days after surgery. X-ray examinations noted that a significantly higher loss in medullary bone and the destruction of cortical bone occurred at the 14th postoperative day in the BCP group **(B)** vs. sham group **(A)**. Pathological sections (hematoxylin-eosin staining) of the left tibia from BCP rats showed that tumor cells were densely packed in the marrow cavity, while polynuclear and heteronuclear cells appeared at the 14th postoperative day **(D)**, vs. sham group **(C)**.

Behavioral responses were the same in each group before the operation. No rat developed a wound infection after the operation to induce BCP model. All rats could freely access food and water. Compared with the sham group, the body weight of rats in the BCP group was gradually lower from day 7 (204.08 ± 7.57 in the sham group vs. 182.97 ± 10.23 in the BCP group, *P* < 0.0001) to day 14 (211.25 ± 7.29 in the sham group vs. 169.00 ± 10.49 in the BCP group, *P* < 0.01) (*n* = 9; **Figure 3A**). The PMWT of rats in the BCP group reduced individually from day 7 (32.62 ± 6.44 in the sham group vs. 9.00 ± 2.19 in the BCP group, *P* < 0.0001) to day 14 (33.15 ± 6.69 in the sham group vs. 3.68 ± 0.95 in the BCP group, *P* < 0.0001) (*n* = 9; **Figure 3B**). The PWTL-cool of rats also gradually decreased from day 7 (285.67 ± 38.64 in the sham group vs. 202.89 ± 21.32 in the BCP group, *P* < 0.0001) to day 14 (297.33 ± 27.78 in the sham group vs. 146.89 ± 12.14 in the BCP group, *P* < 0.0001) (*n* = 9; **Figure 3C**). The PWTL-hot of rats decreased significantly from day 7 (21.78 ± 3.07 in the sham group vs. 16.71 ± 3.80 in the BCP group, *P* < 0.01) to day 14 (22.57 ± 3.61 in the sham group vs. 10.59 ± 2.37 in the BCP group, *P* < 0.01) (*n* = 9; **Figure 3D**). At the end of the experiment (T14), X-ray examination confirmed that a significant loss of medullary bone and the destruction of cortical bone occurred in the cancerous bone (Figure 1B) compared with those in the SHAM bone (Figure 1A). Compared with those in the SHAM bone (Figure 1C), pathological sections (hematoxylin-eosin staining) of the left tibia from BCP rats showed that tumor cells were densely packed in the marrow cavity, while polynuclear and heteronuclear cells appeared on the 14th day post-operatively (Figure 1D). The tibia sections of sham rats did not show any bone destruction or cancer cells.

### The TRPA1 Antagonist A967079 Could Have a Short-Term Influence on BCP

**Figure 2:**
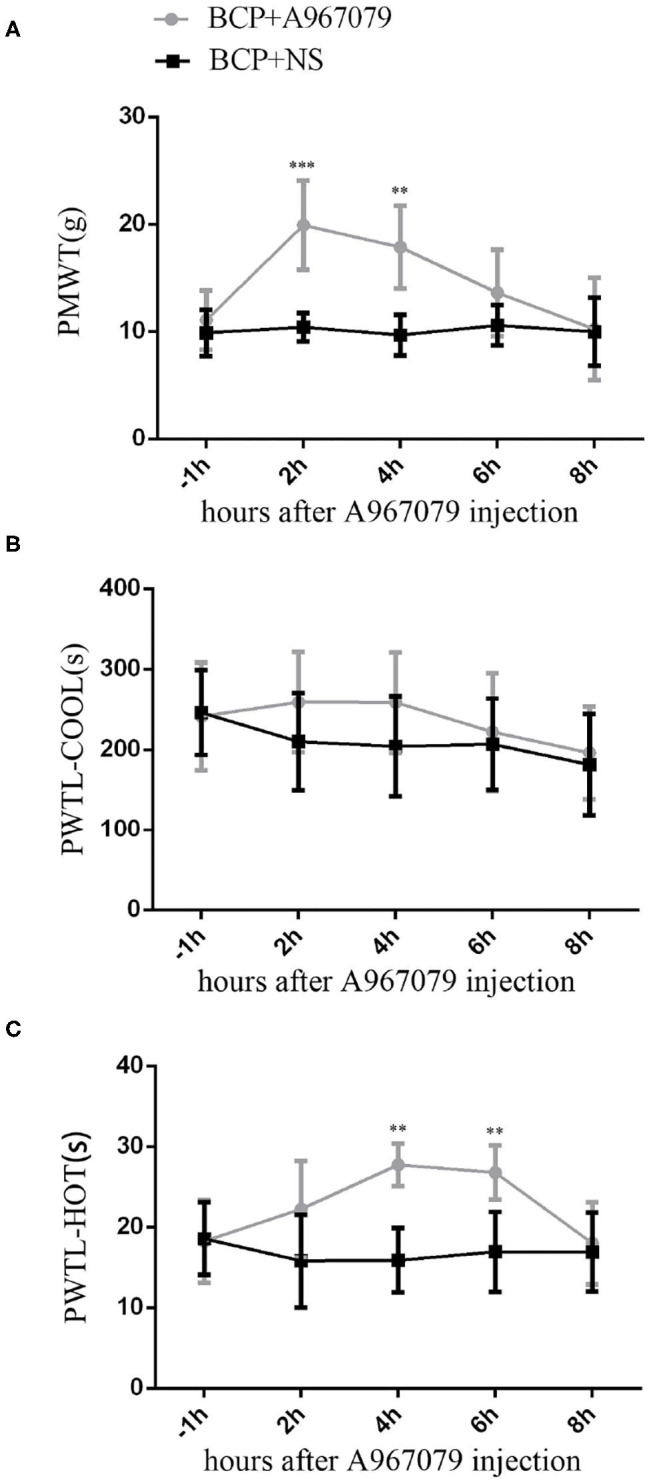
Influence of A967079 (TRPA1 antagonist) on pain-like behavioral response in BCP rats. PMWT and PWTL in the BCP+NS group showed a significant decrease on day 7. Pain-like behavioral responses were detected before and after A967079 was applied once. Data are expressed as the mean ± SD (*n* = 5). **(A)** In the BCP+A967079 group (*n* = 5), PMWT rose significantly from 2 to 4 h. ***P < 0.001, **P < 0.001 vs. BCP+NS group. **(B)** PWTL-cool changed insignificantly, *P* > 0.05 vs. BCP+NS group. **(C)** PWTL-hot increased significantly from 4 to 6 h. **P < 0.01 vs. BCP+NS group.

Since cancer is known to induce serious pain, rats with tumor cells were more sensitive to different stimulus than normal rats. In our experiment, rats in the BCP group showed an obvious response to different stimuli on the 7th day after surgery. Therefore, 10 BCP rats were used. These rats were randomly divided into two groups. In one group, five rats were injected intravenously with A967079 (one of the TRPA1 inhibitors, 10 mg/kg 10 ul) on day 7 (T7) to detect the short-term effect of antagonistic TRPA1 on hyperalgesia induced by a tumor. Compared to the BCP group, A967079 relieved PMWT from 2 h (BCP+NS group: 10.41 ± 1.31 vs. BCP+A967079 group: 19.92 ± 4.16, *P* < 0.001) to 4 h (BCP+NS group: 9.68 ± 1.89 vs. BCP+A967079 group: 17.87 ± 3.85, *P* < 0.01) (*n* = 5; Figure 2A). PWTL-hot also increased from 4 h (BCP+NS group: 15.90 ± 3.97 vs. BCP+A967079 group: 27.74 ± 2.63, *P* < 0.01) to 6 h (BCP+NS group: 16.94 ± 4.99 vs. BCP+A967079 group: 26.80 ± 3.34, *P* < 0.01) (*n* = 5; Figure 2C). However, PWTL-cool improved slightly with (no statistical significance) from 2 h (BCP+NS group: 210.04 ± 60.10 vs. BCP+ A967079 group: 259.20 ± 62.36, *P* > 0.05) to 4 h (BCP+NS group: 204.20 ± 62.18 vs. BCP+A967079 group: 258.74 ± 62.25, *P* > 0.05) (*n* = 5; Figure 2B).

These results suggest that the TRPA1 antagonist can alleviate pain induced by cancer for several hours. They also suggest that TRPA1 could affect BCP conditions.

### TRPA1 Oligodeoxynucleotide Could Affect the Behavioral Response of BCP Consecutively

**Figure 3:**
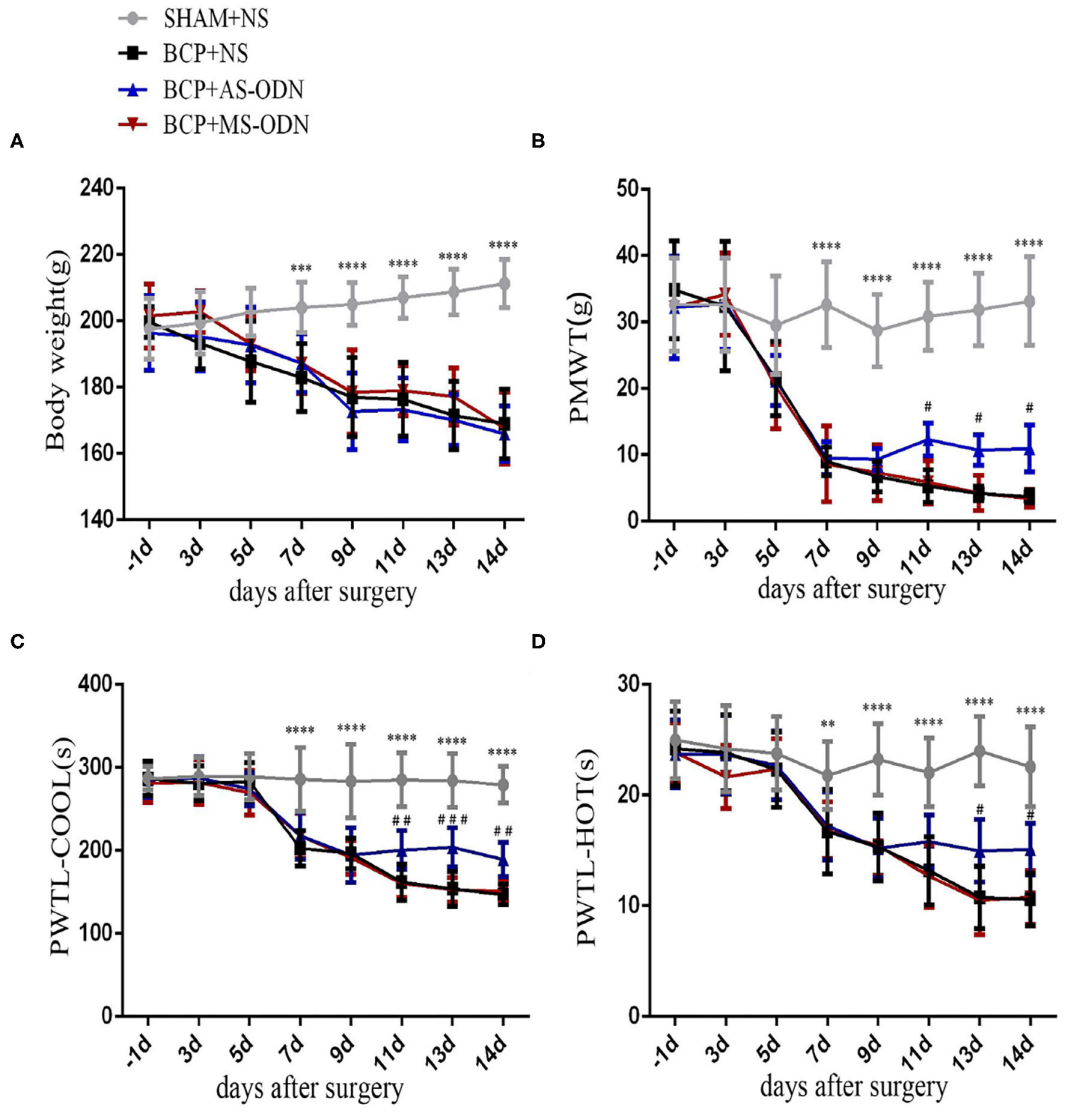
TRPA1 antisense oligonucleotide was applied to detect pain-like responses in BCP rats. Data are expressed as the mean ± SD (*n* = 9). **(A)** In rats with Walker256 cell groups (i.e., the BCP+NS, BCP+AS-ODN, and BCP+MS-ODN groups), body weight significantly decreased from day 7 to 14 after surgery (*n* = 9); ***P < 0.001, ****P < 0.0001 vs. sham at the same time point. **(B)** In the BCP+NS and BCP+MS-ODN groups, PMWT significantly decreased from day 7 to 14 after surgery; **P < 0.01, vs. sham at the same time point. TRPA1 antisense oligonucleotide significantly alleviated PMWT from day 11 to 14 after surgery; ^#^*P* < 0.05 vs. BCP+NS and BCP+MS-ODN at the same time point. **(C)** In the BCP+NS and BCP+MS-ODN groups, PMTL-cool significantly decreased from day 7 to 14 after surgery; ****P < 0.0001 vs. sham at the same time point. TRPA1 antisense oligonucleotide significantly recovered PMTL-cool from day 11 to 14 after surgery;^##^*P* < 0.01, ^###^*P* < 0.001 vs. BCP+NS and BCP+MS-ODN at the same time point. **(D)** In the BCP+NS and BCP+MS-ODN groups, PMTL-hot significantly decreased from day 7 to 14 after surgery. **P < 0.01, ****P < 0.0001 vs. sham at the same time point. TRPA1 antisense oligonucleotide significantly increased PMTL-hot from day 13 to 14 after surgery; ^#^*P* < 0.05 vs. BCP+NS and BCP+MS-ODN at the same time point. PMWT, paw mechanical withdrawal threshold; PWTL-cool, paw withdrawal thermal latency-cool; PWTL-hot, paw withdrawal thermal latency-hot.

AS-ODN and MS-ODN were used for five consecutive days (T9–T13) to determine whether or not TRPA1 could directly influence BCP. The body weights of rats in the BCP+AS-ODN group (T7:187.3 ± 8.79) and BCP+MS-ODN group (T7:187.19 ± 8.96) were similar to those of rats in the BCP+NS group (T7:182.97 ± 10.23) (*P* > 0.05, *n* = 9), which then reduced from day 7 after being injected with Walker256 tumor cells and became lower than those of the sham+NS group (T7:204.08 ± 7.57) (*P* < 0.0001, *n* = 9; Figure 3A). Moreover, compared to BCP+NS group (T11:5.29 ± 2.48, T13:4.18 ± 1.09, T14:3.68 ± 0.95), the PMWT of rats in the BCP+ AS-ODN group mildly increased from T11 (12.29 ± 2.46) to T14 (10.94 ± 3.53) (*P* < 0.05, *n* = 9), while the PMWT of rats in the BCP+MS-ODN group (T11:5.84 ± 3.26, T13:4.24 ± 2.66, T14:3.42 ± 1.36) was similar to that of the BCP+NS group (*P* > 0.05, *n* = 9); however, they were still lower than the sham+NS group (T11:30.86 ± 5.14, T13:31.84 ± 5.46, T14:33.15 ± 6.69) (*P* < 0.0001, *n* = 9; Figure 3B). Moreover, compared to the BCP+NS group (T11:161.78 ± 21.59, T13:153.67 ± 20.68, T14:146.89 ± 12.14), the PWTL-cool of rats in the BCP+AS-ODN group was significantly extended from T11 (200.63 ± 23.42) to T14 (189.11 ± 20.61) (*P* < 0.01, *n* = 9), while the PWTL-cool values of rats in the BCP+MS-ODN group (T11:160.56 ± 17.02, T13:152.78 ± 14.60, T14:150.67 ± 13.56) were similar to those of the BCP+NS group and lower than those of the sham+NS group (T11:285.14 ± 32.38, T13:284.22 ± 32.48, T14:279.33 ± 21.78) (*P* < 0.01, *n* = 9; Figure 3C). Compared to the BCP+NS group (T13:10.76 ± 2.79, T14:10.59 ± 2.37), the PWTL-hot of rats in the BCP+AS-ODN group was extended at T13 (14.97 ± 2.84) and T14 (15.11 ± 2.33) (*P* < 0.05, *n* = 9), while the PWTL-hot values of rats in the BCP+MS-ODN group (T13:10.50 ± 3.09, T14:10.77 ± 20.45) were similar to those of the BCP+NS group; however, they were still shorter than those of the sham group (T13: 23.98 ± 3.14, T14:22.57 ± 3.61) (*P* < 0.01, *n* = 9; Figure 3D).

### TRPA1 Expression Among Different Groups

**Figure 4:**
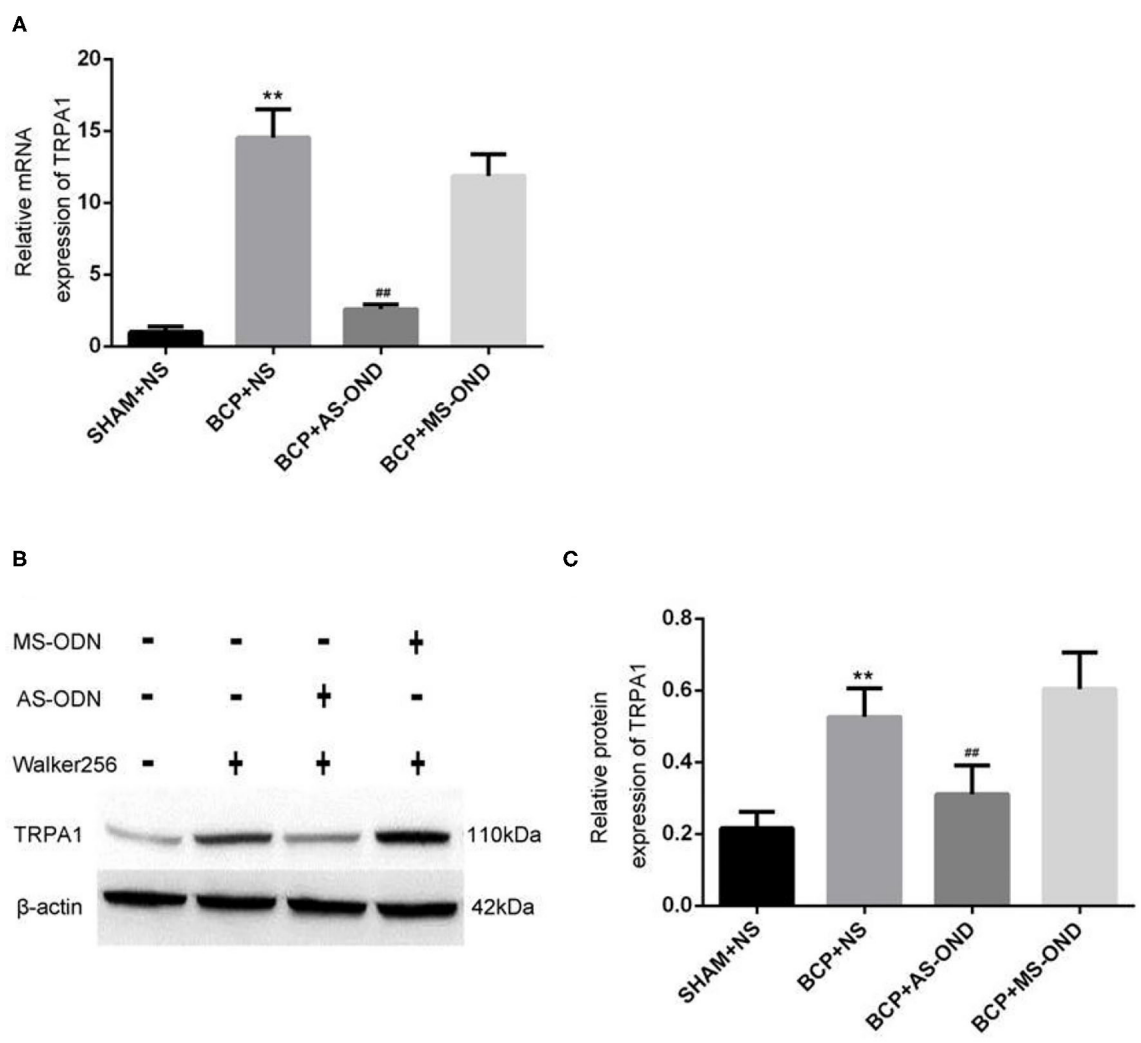
Effect of TRPA1 antisense oligonucleotide (AS-ODN) on BCP rats. **(A)** Data are expressed as the mean ± SD (*n* = 4). mRNA expression in the dorsal root ganglion of BCP rats; **P < 0.01, vs. sham+NS; ^##^*P* < 0.01 vs. BCP+NS; *P* > 0.05 BCP+MS-ODN vs. BCP+NS. **(B)** Expression of TRPA1 protein detected in each group by western blot assay. **(C)** Data are expressed as the mean±SD (*n* = 5). Relative mRNA expression of TRPA1 is expressed as the optical density ratio of TRPA1/β-Actin (*n* = 9). **P < 0.01 vs. sham+NS; ^##^*P* < 0.01 vs. BCP+NS; *P* > 0.05 BCP+MS-ODN vs. BCP+NS.

Quantitative RT-PCR was applied to investigate the role of TRPA1 on mRNA in nociceptive neurons. Compared to the sham+NS group (1.66 ± 0.32), the level of TRPA1 mRNA expression significantly increased in the DRG of BCP rats (14.55 ± 1.87) at T14 (*P* < 0.01). While the TRPA1 mRNA expression was significantly reversed in the DRG of rats in the BCP+AS-ODN group (3.29 ± 0.34) (*P* < 0.01; Figure 4A), there was no significant difference between the BCP+MS-ODN group and the BCP+NS group. These results indicate that BCP would be relieved by inhibiting TRPA1. Furthermore, western blot was used to detect the protein of TRPA1 in injured DRG. Compared to sham+NS group, we found that the level of TRPA1 protein expression was higher in the BCP model rats (*P* < 0.01). However, this expression could be reversed when TRPA1 AS-ODN was applied. While the protein expression of TRPA1 in the BCP+MS-ODN group was slightly higher than that of the sham group, no significant difference was observed between them (*P* > 0.05; Figures 4B,C). This also supports the notion that TRPA1 was involved in BCP.

## Discussion

The results suggest that TRPA1 is involved in regulating the occurrence of BCP. This study mainly verified the role of TRPA1 in bone cancer transmission by antagonizing and knocking down TRPA1 expression. First, intravenously injected TRPA1 antagonist A967079 can rapidly relieve BCP and hot hyperalgesia, which is consistent with previous research [27]; however, it is not effective against cool hyperalgesia. Additionally, TRPA1 antisense oligonucleotide (AS-ODN) was injected intrathecally by a mini-osmotic pump for 5 consecutive days to detect pain-like response after TRPA1 was knocked out, and it was found that knocking out TRPA1 can reduce mechanical hyperalgesia and thermal (cool and hot) hyperalgesia. Therefore, it can be concluded that the TRPA1 channel may be involved in mediating mechanical and thermal hyperalgesia in rat BCP models.

With the improvement of diagnosis and treatment, the survival time of cancer patients has gradually increased [28, 29]. However, 75% of advanced cancer patients still feel pain, which affects their quality of life. Especially in breast cancer and prostate cancer, 60–80% of patients have bone metastases [28]. BCP has complex pain properties, including spontaneous pain, exercise-induced pain and persistent pain. The first two usually manifest as sudden bursts of pain that are difficult to effectively control with conventional analgesics [29]. Various studies have shown that cancer pain is a complex pain that involves peripheral nervous system activity, spinal cord transmission and related molecules; however, it is ultimately controlled by brain tissues throughout the pain network [10, 11]. In recent years, studies have found that TRPA1, as a member of the TRPs family, can regulate and conduct inflammatory pain and neuropathic pain; furthermore, it can also sense temperature changes below 17°C in the environment [30–33]. Notably, Honda [34] suggested that TRPA1 mediates cold facial hyperalgesia in rats. Additionally, Schwartz et al. [35] also showed that TRPA1 mediates pain associated with acute pancreatitis. Moreover, Nassini et al. [36] showed that TRPA1 mediates oxaliplatin-induced mechanical hyperalgesia and cold hyperalgesia in rats. Furthermore, Elizabeth [37] and other studies also showed that TRPA1 mediates arthritis pain caused by cold environmental stimulation. Furthermore, other subtypes in the TRPs family were reported to be involved in regulating tumor-induced nociceptive pain, such as transient receptor potential vanilloid 1 (TRPV1), transient receptor potential vanilloid 3 (TRPV3), transient receptor potential vanilloid 4 (TRPV4), and transient receptor potential melastatin 8 (TRPM8) [35, 38]. However, there are few reports on whether TRPA1 is involved in regulating the complexity of cancer pain and the occurrence and development of temperature sensitivity; thus, further research is required.

In this study, Walker256 tumor cells were injected into the tibia and bone marrow cavity of SD rats according to the method described by Shenoy [21]. The general state and pain-related behavioral responses of rats were measured before surgery and every other day after tumor cell inoculation. Notably, 7–14 days after the model was established, the mechanical pain threshold of rats—even the feeling caused by light contact—gradually decreased with tumor growth. Simultaneously, the dwell time of BCP rats on hot and cold plates was observed. The results of these observations suggest that rats experience cold and hot hyperalgesia as a tumor grows, which enriches the study of temperature-related sensations of BCP. The successful establishment of this model provides a good basis for the subsequent evaluation of trends related to pain-related behaviors and determining the timing of drug intervention.

This study not only found that the administration of TRPA1 antagonists can alleviate BCP and cold pain sensitivity in a short time but also observed that knocking down this gene can reduce BCP cold and hot hyperalgesia. According to different experiments, the synthesized mistranslation oligonucleotide chain is a missense code synthesized based on antisense oligonucleotides and has no practical effect [39–41]. Notably, it is used to control antisense oligonucleotides. In the experiment, the effect of missense oligoglycinic acid on behavior, relative mRNA and protein expression was not the opposite of normal saline, which contrasted the role of antisense oligoglycinic acid.

Antoniazzi et al. [42] also showed that blocking TRPA1 can alleviate mechanical and cold hyperalgesia in tumor mice—which is consistent with the present study—while its inability to alleviate thermal hyperalgesia is contrary to this study and deserves to be further study. Furthermore, the research of Maqboul et al. [43] on TRPA1-mediated tumor-induced mechanical hyperalgesia is consistent with this study; however, the model of Ahmad et al. involved injecting tumor cells into the perineal nerve sheath of rats. While differences exist between these two studies, both suggest that tumor-induced pain is related to TRPA1. The specific mechanism of action includes different ion channels sensing nociceptive stimuli in the environment and converting them into electrical signals while producing pain-like behaviors. Previous studies have shown that TRPA1 and TRPV1 often mediate inflammatory and neuropathic pain. However, TRPV1 expression was abnormal in one BCP study [12]. According to previous reports, TRPA1 is a common pathway for the action of multiple chemically harmful agonists that can be directly activated by environmental cinnamaldehyde (cinnamon compounds), AITC (mustard oil compounds), allicin (garlic compounds), formalin (environmental pollutants) as well as *in vivo* bradykinin, while NGF and BDNF (among others) are indirectly activated. In addition to the inflammatory, neuropathological and ischaemic factors in BCP, tumor bone metastasis itself can activate primary nociceptiveness and change the osteogenesis/osteoclast balance. Among them, BDNF and TNF produced in bone tumor metastasis metabolism-α, MCP-1 and CCR2 can activate TRPA1 [3, 44]. Notably, the results reported in the literature are consistent with the results of the present study.

Although useful, this study has certain limitations. First, TRPA1 is sensitive to cold stimuli below 17°C, while and both cold and hot pain change in BCP. Thus, determining whether temperature-dependent ion channels are involved is worthy of further study. Second, TRPA1 can be activated by a variety of ions, and its related pathways—which mediate BCP—deserve further exploration. Third, a time course of bone cancer histological characterization of the BCP model should have been performed in this study. Fourth, A967079, TRPA1 antagonist, should be given in multiple doses to allow more significant and durable drug effect. Finally, determining whether TRPA1 is related to tumorigenesis and development is also worthy of further study. Future work should utilize models to study the aforementioned questions.

In summary, the TRPA1 channel mediates mechanical allodynia and thermal hyperalgesia in a rat BCP model and can be used as a new target for alleviating BCP.

## Data Availability Statement

The data used to support the findings of this study are available from the corresponding author upon request.

## Author Contributions

HZ and LX contributed to the design of the study and the review of the literature. LF, XH, WZ, and QL participated in data collection, analysis, and drifting of the manuscript. All authors have read and approved the manuscript.

## Conflict of Interest

The authors declare that the research was conducted in the absence of any commercial or financial relationships that could be construed as a potential conflict of interest.
